# Sex or poison? Genetic pest management in the 21st century

**DOI:** 10.1186/s12915-023-01785-x

**Published:** 2023-12-29

**Authors:** Luke Alphey

**Affiliations:** 1 Department of Biology, University of York, York, YO10 5DD UK; 2 York Biomedical Research Institute, University of York, York, YO10 5DD UK

## Abstract

**Advances in genetic technology have made possible a suite of genetics-based methods for controlling pests of importance to public health, agriculture and conservation.**

## Genetic pest management

Pests do enormous damage to human and animal health, to agriculture and to biodiversity, with mosquitoes transmitting pathogens, insect larvae eating crops or invasive rodents threatening the last island refuges of endangered birds. This commentary focuses on insects, particularly mosquitoes. However, most considerations apply equally to other pest species. Genetic pest management (GPM) is the use of genetics to control pests through mating of modified pests with their wildtype counterparts [1]. This allows heritable traits to be transferred (“introgressed”) into the wild pest population. In principle, any sexually reproducing pest species can be targeted.

The aim is to reduce harm done by the pest population. with typical intended outcomes overwhelmingly falling into two types: population suppression and population modification. For population suppression, one would introgress fitness-reducing traits, such as lethality or sterility, leading to reduction in the numerical size of the pest populations if spread into the target population at sufficiently high frequency. Population modification aims to reduce the harm done by the pest without large changes in the numerical size of the pest population, for example by reducing the ability to transmit disease (“vector competence”) of modified mosquitoes. If such traits, or the DNA sequences encoding them, can be sustained at sufficiently high allele frequency in the target population then the desired harm-reduction outcome should be achieved, by reduction in the number of pests or by reduction in the per-pest harm.

As well as selecting and engineering the desired trait, a key aspect that is the target of much current research is how to “spread into the target at sufficiently high frequency”, of which more below.

The various GPM approaches share several characteristics which distinguish them from other approaches such as the use of chemical pesticides.

- *Species-specificity*: Mating-based delivery makes GPM exquisitely species-specific, since the modified insects will mate only with their own species (or, sometimes, very closely related species). This specificity is highly desirable from an environmental perspective. However, if many pest species are simultaneously present, a more broad-spectrum approach may be preferred.
- *The insects will be released over a wide area*: This is not an ‘individual-based’ approach such as vaccination, or bednets — which have community-level benefits but are applied at the level of the individual and may therefore be based on individual consent. In contrast, area-wide interventions require community-level consent, as typical for other larger infrastructure projects.
- *Equitable*: All individuals in the treated area are equally protected, irrespective of wealth, education, or other characteristics. This is commonly not the case for individual-based interventions.
- *Access to hard-to-reach/cryptic populations*: In contrast to chemical pesticides, modified insects will actively seek out their wild counterparts for mating. In contrast, conventional methods often struggle to reach cryptic or low-level populations.

GPM therefore has distinctive features that are highly desirable in many, but not all, settings. While occasionally advocated as ‘stand-alone’ or even ‘silver bullet’ methods by enthusiasts, they are better considered as valuable new tools within an integrated pest management system.

## GPM in the 20th century

GPM has a relatively long history, starting with the classic Sterile Insect Technique (SIT). Pest insects were mass-reared, sterilized — usually by irradiation — and released to mate with the wild pests [2]. Pest females who mated a sterile male would have fewer offspring than otherwise, and so if enough wild females could be induced to mate sterile males the target population would tend to decline. This typically requires that most of the pest females’ matings are with sterile males, and therefore that the sterile male population considerably outnumbers the wild male population. Since for relevant pest insects, the sterile males do not live very long, this requires large, frequent releases of sterile males and a substantial rearing and release infrastructure to support such programs. Nonetheless, very large and successful programs were conducted against specific pests, including the Mediterranean fruit fly (Medfly) and other tephritids. Most dramatically, the New World screwworm was eliminated from North and Central America by a program centred on large-scale release of sterile screwworm flies, starting in the USA (Florida 1957, Texas 1962), moving to Mexico in the 1980s and to Panama in the 1990s, where ongoing release in a barrier program prevents reinvasion from further south. This highlights one of the attractive features of sterile-male methods — since the ratio of sterile:wild males increases as the target population declines, the method becomes more effective over time, and is particularly good at removing the last, small, residual population.

Most programs used essentially wildtype pest insects, albeit with some inadvertent changes associated with colonization and mass-rearing. Since released sterile females tend to ‘distract’ co-released sterile males so they mate fewer wild females, male-only releases are much preferred. Furthermore, for some species even sterile females are damaging — for Medfly, oviposition damages the fruit even if the eggs do not hatch; for mosquitoes only females bite — so male-only releases are strongly preferred to avoid the possibility of harm from the sterile insects themselves. In a tour-de-force of classical genetics, researchers at the IAEA developed sophisticated “genetic sexing strains” of Medfly in which females could be eliminated simply by heat treatment [2].

## Rapid advances in the past two decades

The 20 years since the first issue of BMC Biology have seen a dramatic expansion of prospects, possibilities, and field use of GPM. Insect synthetic biology has brought major improvements; an engineered mosquito first described in BMC Biology in 2007 [3] entered field trials from 2009, around 1 billion males were released by Oxitec in successful trials and programmes before the strain was replaced by an improved version.

Oxitec’s approach is still recognisably a sterile-male method, albeit using synthetic biology to replace radiation, and later to provide other desirable traits such as sex-separation, and new possibilities for community participation. Other sterile-male methods were developed, together with Oxitec’s synthetic biology approach the most prominent is the use of *Wolbachia. Wolbachia* is a diverse species of bacterium, various strains of which infect a range of arthropods. Rather than being infectious in the normal sense (horizontal transmission), it is almost exclusively vertically transmitted, specifically from an infected mother to her offspring. Many strains are reproductive parasites, manipulating their host’s reproductive biology to promote their own transmission. This can take various forms; one is to arrange that infected males are fertile only with infected females. Correspondingly they are sterile with wild females — and can be used in a sterile-male program! This requires that the ‘sterile’ males have a suitable strain of *Wolbachia* that the wild pest population does not, which usually means artificial transfer of the *Wolbachia* from another insect species. Crucially, it also requires that no infected females are released, since they are fertile with both wild-type and infected males — and so are all their female offspring. Though the need for stringent sex-separation is a significant disadvantage, there are some compensating advantages, for example the method seems to produce relatively high-quality, competitive ‘sterile’ males. Significantly, though the artificially trans-infected strain has a novel heritable trait as a product of modern biotechnology, such strains have not been considered “Genetically [or “Living”] Modified Organisms” (GMOs/LMOs) in several countries, avoiding the broader controversy and stringent regulation around the use of genetic technologies in the environment.

Each of these new sterile-male methods represents a considerable advance over the previous state of the art. However, they all need large, frequent, long-term releases. Though feasible and economic for a surprising number of pest insects, it would clearly be desirable from an economic perspective to achieve the desired effect from release of fewer modified insects. This may not be an unalloyed benefit, as it implies much greater persistence of the modification in the field — really the only means to reduce the frequency of releases. For a heritable genetic modification, persistence, reversibility and controllability are all intimately related.

Though the novel heritable traits are beneficial for humans, they are likely not so for the pest, and so will be rapidly eliminated from target populations by natural selection — unless it can be arranged otherwise. Systems in which DNA sequences spread through a population despite conferring a fitness cost on carriers, are widespread in nature — “gene drives”, also known as selfish DNA [4]. It has long been proposed that such systems might be used to spread desirable traits [5], but the technology to do this has only recently become available. Austin Burt’s seminal paper [6] described ‘homing’ drives — still the leading approach for synthetic gene drives, though with many variants; development was greatly accelerated by the discovery of CRISPR/Cas9. However, despite remarkable laboratory progress, the only gene drive to have achieved field use is instead based on *Wolbachia*.

As outlined above, *Wolbachia* acts as a gene drive, imposing a fitness cost on non-carriers relative to carriers that allows this heritable element to spread through target populations (uninfected females are infertile with infected males, but infected females are fertile with both infected and uninfected males, uninfected females therefore suffer a fitness cost that infected females do not, and which increases as the proportion of infected males increases). Some strains of *Wolbachia* inhibit replication of some viruses in their host, the best-known of these being *w*Mel from *Drosophila melanogaster*. Remarkably, *w*Mel retains its virus-blocking property when artificially transferred to the mosquito *Aedes aegypti*, while also showing strong gene drive properties. This has been spread into field populations — the initial introduction requires large releases, as with sterile males, but once established in a population the infection may persist for years without further releases. A trial of this “gene drive plus reduced-vector-competence” system in Indonesia reduced symptomatic dengue incidence by 77.1% (95% CI, 65.3 to 84.9), a remarkable result [7]. Furthermore, at least in the lab, *Wolbachia* protects against several other viruses transmitted by this mosquito — though the degree may vary from one virus to another and some insect-specific viruses are enhanced rather than suppressed. Still, while the long-term stability/evolution of this complex mosquito-bacterium-virus-human system remain to be clarified, results so far have been extremely encouraging.

## Where will we be in another two decades?

We can confidently predict that the successful approaches of the past two decades — enhanced sterile-male methods, and *Wolbachia* gene drive — will continue to make further advances in the field. Indeed, one could argue that take-up has been rather slow, considering the demonstrated effectiveness of these methods. Perhaps this will accelerate with greater familiarization, though funding is always problematic for these neglected tropical diseases.

For malaria, conventional methods have substantially reduced morbidity and mortality, but progress seems to have stalled. Indeed, increasing resistance to insecticides and anti-malarial drugs challenges even the progress made so far. Sterile-male methods may be uneconomic for the major rural vectors, and *Wolbachia* has shown little promise so far in *Anopheles*. However, the key malaria vectors, especially *Anopheles gambiae*, have been a major target for synthetic gene drives. Target Malaria has led the way (e.g. [8]), but there are now several major groups and consortia working in this area (e.g. [9]). I think within the next 20 years we will see multiple field trials of gene drives — or components thereof, since it is difficult to conduct a limited trial of a highly invasive gene drive — and deployment of at least one, more likely several. Crucially, “gene drives” are highly diverse, both in nature and for synthetic biologists, and may be, by design, more or less invasive, more or less persistent, and directed at either population suppression or population replacement. Different communities may make different choices as to which of these properties they prefer; it is the role of developers to provide a range of practical options to allow those choices to be realised.

Development of GPM methods for public health will facilitate other applications. Invasive species are a huge problem for biodiversity and conservation; the species-specific nature of GPM is highly desirable in sensitive ecologically-sensitive areas. Invasive rodents threaten a wide range of island habitats — mouse genetics is relatively well understood, and mouse gene drives are being developed, for example based on the natural *t* haplotype system [10]. Laboratory progress has recently been good; given the large and pressing nature of the problem one might anticipate field trials and, if successful, larger-scale use, well within the next 20 years.

Though I have focused on the pest genetics, implementing a GPM program requires a wide range of other technologies, including mass-rearing, release, surveillance, etc. Many of these are ripe for improvement, and each could further improve the cost-benefit of GPM approaches.

These are exciting times for GPM — when founding Oxitec in 2002, the field looked ‘uncluttered’ beyond the large and mostly government-run SIT programmes. Now there are a dizzying array of start-ups and larger companies involved in GPM methods, as well as academic groups — we can confidently expect that the next 20 years will provide even more progress and human and environmental benefits than the last.

## Data Availability

Not applicable.
